# Threatened and Endangered Subspecies with Vulnerable Ecological Traits Also Have High Susceptibility to Sea Level Rise and Habitat Fragmentation

**DOI:** 10.1371/journal.pone.0070647

**Published:** 2013-08-05

**Authors:** Allison M. Benscoter, Joshua S. Reece, Reed F. Noss, Laura A. Brandt, Frank J. Mazzotti, Stephanie S. Romañach, James I. Watling

**Affiliations:** 1 University of Florida, Fort Lauderdale Research and Education Center, Davie, Florida, United States of America; 2 Valdosta State University, Department of Biology, Valdosta, Georgia, United States of America; 3 University of Central Florida, Department of Biology, Orlando, Florida, United States of America; 4 United States Fish and Wildlife Service, Davie, Florida, United States of America; 5 United States Geological Survey, Southeast Ecological Science Center, Davie, Florida, United States of America; University of Western Ontario, Canada

## Abstract

The presence of multiple interacting threats to biodiversity and the increasing rate of species extinction make it critical to prioritize management efforts on species and communities that maximize conservation success. We implemented a multi-step approach that coupled vulnerability assessments evaluating threats to Florida taxa such as climate change, sea-level rise, and habitat fragmentation with in-depth literature surveys of taxon-specific ecological traits. The vulnerability, adaptive capacity, and ecological traits of 12 threatened and endangered subspecies were compared to non-listed subspecies of the same parent species. Overall, the threatened and endangered subspecies showed high vulnerability and low adaptive capacity, in particular to sea level rise and habitat fragmentation. They also exhibited larger home ranges and greater dispersal limitation compared to non-endangered subspecies, which may inhibit their ability to track changing climate in fragmented landscapes. There was evidence for lower reproductive capacity in some of the threatened or endangered taxa, but not for most. Taxa located in the Florida Keys or in other low coastal areas were most vulnerable to sea level rise, and also showed low levels of adaptive capacity, indicating they may have a lower probability of conservation success. Our analysis of at-risk subspecies and closely related non-endangered subspecies demonstrates that ecological traits help to explain observed differences in vulnerability and adaptive capacity. This study points to the importance of assessing the relative contributions of multiple threats and evaluating conservation value at the species (or subspecies) level when resources are limited and several factors affect conservation success.

## Introduction

Assessing the vulnerability (sensitivity+exposure to threats) and adaptive capacity (ability to adjust to threats) of species helps identify the most important factors affecting species survival and prioritize limited conservation resources. In addition to pressures such as habitat fragmentation [1], invasive species [2], disease [3], and overexploitation [4], climate is a critical factor affecting the survival and distribution of species. Climate affects biodiversity at multiple spatial and taxonomic scales, and recent climate change is already linked to alterations in species phenology, survival, and distribution [5], [6], [7], [8]. Climate change may present unique challenges to threatened and endangered taxa (hereafter referred to as endangered), because these species often exist in small populations, have limited genetic variation, and may have ecological traits that make them vulnerable to rapid environmental change. It is cost-effective to prioritize conservation efforts towards species and communities that have a relatively high probability of conservation success, and provide ecological, evolutionary, social, or economic value.

Florida is a national hotspot of endemism [9], [10], making biological conservation significant at local, national, and broader levels. There are also many taxa at risk of extinction, and only three other states in the country have more federally endangered taxa than Florida (Hawaii, California, and Alabama). Of the 572 endangered animal taxa listed on the federal register in the United States, 53 occur in Florida (approximately 10% of all listed taxa). Of these 53 federally listed taxa, 23 are designated as subspecies (approximately 43% of Florida’s listed taxa), and 19 of the 23 subspecies are endemic (unique) to Florida [11]. Understanding the vulnerability of endangered subspecies in this region to factors such as climate change and habitat fragmentation may help reduce extinction rates for Florida’s endangered vertebrates.

Numerous pressures affect native species persistence in Florida, including climate change. Florida is particularly vulnerable to sea level rise, with about 10% of its land area less than 1 m above current sea level [12]. Most of the Florida Keys are predicted to be inundated or drastically altered under a sea level rise scenario of 0.6 m [13], [14], and substantial tracts of the Everglades, other low-lying coastal areas, and barrier islands across the state will be submerged or severely modified with 1 m of sea level rise [15]. Many areas that are not inundated by increased sea level will be susceptible to storm surges, flooding, erosion, and other risks [5]. Although Florida’s climate is predicted to warm less than other regions in North America [5], [16], a climate inventory over the past 35 to 108 years indicated Florida is experiencing greater climate extremes, with trends of increased summer and fall maximum temperatures and decreased winter and spring minimum temperatures [17]. The intensity of tropical storms is also predicted to increase, although frequency may decrease [5], [18]. Given the link between extreme climate events and the decline of local populations [13],[19], an increase in frequency or intensity of extreme climate may threaten endangered species that exist in small fragmented populations.

High human population growth rates and non-native species invasions also threaten native fauna of this region. Florida’s human population growth rate was the third highest in the United States between 2000 and 2010 [20]. Florida also has a high incidence of non-native plant and animal species [21], [22]. These pressures coupled with high biodiversity and limited conservation resources make setting conservation priorities extremely important.

Vulnerability assessments are a valuable tool that aid in prioritizing conservation efforts through the evaluation of threats and their impacts on species and communities. The Standardized Index for Vulnerability and Value Assessment (SIVVA) is a novel tool [23] that is useful for evaluating vulnerability, adaptive capacity, and conservation value of species and communities. It provides several improvements over previous vulnerability assessments, such as the explicit incorporation of sea level rise and the ability to account for uncertainty in the assessment process. It allows for inclusion of the ecological, evolutionary, and economic value of taxa, and offers a flexible and modular approach applicable to a broad range of taxonomic groups, including terrestrial, freshwater aquatic, and marine species. SIVVA allows for the evaluation of multiple interacting threats, both independently and collectively, to measure vulnerability and adaptive capacity, allowing for a more comprehensive assessment that is particularly useful for setting conservation priorities in regions that face numerous threats to biodiversity.

In this study we incorporated a multi-step approach to evaluate vulnerability and adaptive capacity of federally endangered subspecies in Florida. We focused on subspecies pairs that differed in endangered status, because their phylogenetic relatedness provided the opportunity to ask questions about ecological traits related to adaptation and vulnerability. Because each subspecies in the taxon pair represented a closely related but distinct population, potential variation in ecological traits may help explain the relative ability to respond to environmental change. This framework allowed for a robust test to examine potential differences in traits for endangered taxa, especially given that controlled experiments on these subspecies are not feasible. Although comparing traits among related taxa and accounting for phylogenetic similarity is not uncommon in ecological studies, we are unaware of other regional-scale studies designed to investigate trait differences among many pairs of closely related taxa (subspecies) that vary in conservation status. The target group for this study included all of the federally endangered subspecies located in Florida.

We examined reproductive output, home range size, dispersal ability, and survival because they can affect population persistence and extinction risk [24], [25], [26]. Our approach coupled SIVVA with in-depth literature surveys of ecological traits to evaluate vulnerability and adaptive capacity of endangered subspecies under future scenarios of sea level rise, habitat fragmentation, and climate. Because endangered taxa often have restricted ranges, small population sizes, and reduced genetic variation, we expected endangered subspecies to demonstrate higher vulnerability and lower adaptive capacity compared to closely related, non-endangered subspecies. We also developed four specific *a priori* hypotheses related to vulnerability criteria important in Florida, and predicted that endangered subspecies would have greater vulnerability to sea level rise, habitat fragmentation, and altered temperature and precipitation regimes. Lastly, we expected that endangered subspecies would have higher vulnerability and decreased adaptive capacity because of lower reproductive output, greater home range size, shorter dispersal, and lower survival rates.

## Materials and Methods

### 1. Study Taxa

Study taxa comprised 12 taxon-pairs (n = 8 mammals, n = 4 birds). Each pair consisted of a federally endangered subspecies and a closely related non-endangered subspecies in the same parent species group (in a few cases, two non-endangered subspecies were used for comparison; Table 1). Although the target group included taxon-pairs encompassing all of the federally threatened and endangered subspecies in Florida (n = 23 animals, n = 2 plants), analyses were conducted on 12 taxon-pairs due to limited data for 13 taxon-pairs. Scientific nomenclature for endangered subspecies followed the U.S. Fish and Wildlife Service (USFWS) Endangered Species Program [11]; nomenclature for non-endangered subspecies followed the Integrated Taxonomic Information System (ITIS) [27] and supplemental literature when indicated. Although there were a few cases of disputed taxonomy (Table 1), all federally endangered subspecies represent distinct populations that do not interact with the non-federally endangered subspecies. Non-endangered subspecies were chosen based on information availability, with preference given to those with the greatest geographic similarity.

**Table 1:** Study species.

Federally listed subspecies	Common name	Federal status	Non-listed subspecies
**Mammals**			
*Neotoma floridana smalli*	Key Largo woodrat	Endangered	*floridana*
*Odocoileus virginianus clavium*	Key deer	Endangered	*virginianus*
*Oryzomys palustris natator* a	Silver rice rat	Endangered	*natator*, *palustris* b
*Puma concolor coryi*	Florida panther	Endangered	*couguar* c
*Peromyscus gossypinus allapaticola*	Florida salt marsh vole	Endangered	*gossypinus*
*Peromyscus polionotus niveiventris*	Southeastern beach mouse	Threatened	*rhoadsi*, *subgriseus*
*Peromyscus polionotus phasma*	Anastasia Island beach mouse	Endangered	*rhoadsi*, *subgriseus*
*Sylvilagus palustris hefneri*	Lower Keys marsh rabbit	Endangered	*paludicola*
**Birds**			
*Ammodramus maritimus mirabilis*	Cape Sable seaside sparrow	Endangered	*maritimus, peninsulae*
*Ammodramus savannarum floridanus*	Florida grasshopper sparrow	Endangered	*pratensis*, *perpallidus*
*Polyborus plancus audubonii*	Audubon crested caracara	Threatened	*cheriway* d
*Rostrhamus sociabilis plumbeus*	Everglade snail kite	Endangered	*sociabilis*

Study species comprised 12 taxon-pairs, each included a federally threatened or endangered subspecies and a closely related non-listed subspecies in the same parent species group (in some cases, two non-listed subspecies were used for comparison). Scientific nomenclature for listed subspecies followed the U.S. Fish and Wildlife Service Endangered Species Program [11]; nomenclature for non-listed subspecies followed the Integrated Taxonomic Information System [27] and supplemental literature when indicated.

a The endangered taxon *Oryzomys palustris natator* [11] is also referred to as *Oryzomys argentatus* [66].

b Subspecies taxonomy for *O. palustris* has undergone several revisions. For this study, we compared the federally endangered population (*O*. *p. natator* aka *O. argentatus*) to two mainland subspecies of *O. palustris* located in Florida and the southeastern United States. According to [66], *O. p. natator* is a subspecies that occurs in central Florida; it does not interact with the endangered population.

c Subspecies taxonomy for *Puma concolor* has undergone several revisions. According to [46], *P*. *c*. *couguar* refers to cougars throughout North America.

d *Polyborus plancus audubonii* [11] is included as a sub-population of the species *Caracara cheriway* (northern crested caracara) according to ITIS [27] nomenclature. We compared the *P*. *p*. *audubonii* population to other non-interacting individuals with the most geographic similarity in the *C. cheriway* complex.

### 2. Vulnerability Assessments (SIVVA)

Vulnerability assessments using the SIVVA framework were conducted for 12 endangered subspecies and their closely related non-endangered subspecies (n = 23 total assessments; *Peromyscus polionotus phasma* and *Peromyscus polionotus niveiventris* have the same parent species, *Peromyscus polionotus*; Table 1). The SIVVA framework consists of four modules: vulnerability (sensitivity+exposure to threats), adaptive capacity (ability to adjust to threats), conservation value, and information availability. Each module contains a set of criteria (n = 30 total SIVVA criteria) that describe key threats and factors relevant to conservation planning. For example, the vulnerability module includes 12 different criteria describing potential threats to species persistence, including sea level rise, habitat fragmentation, and altered temperature and precipitation (Table S1).

Each taxon was assessed independently by two experts with knowledge regarding the taxon of interest. Experts were provided with detailed taxon range maps, projections (e.g., sea level rise, human population growth), and a summary of published literature for the taxon. The accuracy of expert opinion greatly improves when it is coupled with consultation of relevant literature [28], [29]. For each assessment, the taxon was scored on a continuous scale from zero to six for each of 30 criteria. In general, higher scores corresponded to higher vulnerability, lower adaptive capacity, greater conservation value, and greater information availability (e.g., we assumed greater information availability enhances the probability of conservation success). For example, vulnerability criterion scores of one and two corresponded to positive responses to projected environmental change (one was most positive), a score of three corresponded to no effect, and scores of four, five, and six indicated increasingly negative responses to the environmental threat. Scores reflect expert opinion, but have quantitative guidelines, for example, a score of six for vulnerability to sea level rise corresponded to “50% or more of known range being inundated by 1 m of sea level rise by 2100.” For all criteria, a score of zero indicated insufficient information to evaluate the criterion. Detailed descriptions of score metrics were provided with each criterion, and although the specific metrics related to each score varied depending on the criterion, the relative scale was constant.

Summary scores were calculated for each module as the total number of points divided by the total number of possible points (Table S1). Note that a higher adaptive capacity score equates to reduced adaptive potential. We also applied a pre-determined weighting scheme that reflected the relative importance of the criteria for terrestrial vertebrate taxa [23]. Weights were randomly permuted 1000 times to assess the influence of the weighting scheme on each module score for each taxon. The level of expert certainty was also incorporated via a checked box next to criterion scores with low confidence. To account for the uncertainty, values of zero or one were added or subtracted from each checked criterion (while maintaining a score range of 1–6), and each module score was recalculated using 1000 Monte Carlo simulations. From these simulations, we created 95% confidence intervals around each module score that summarized potential deviations in the overall score resulting from potential variability in criterion scores due to assessor uncertainty. For a more detailed description of SIVVA, refer to Reece and Noss [23].

### 3. Ecological Traits

We gathered information on ecological traits from published literature for 12 taxon-pairs and 4 trait categories: litter or clutch size, home range size, dispersal distance, and annual adult survival (Dataset S1). Observations from published literature were classified as independent if they contained data without overlapping individuals, locations, and/or time periods. Within each taxon-pair x trait combination we controlled for factors such as gender, age class (e.g., juvenile vs. adult), sample duration (e.g., monthly vs. annual survival), and measurement unit. When applicable, data were converted to maintain constant sample units for each taxon-pair x trait combination (e.g., home range data reported as hectares were converted to km^2^). We obtained information for at least one trait for each of the 12 taxon-pairs. Territory size was used as the home range metric in *Ammodramus* taxa. We also compiled data that quantified geographic distance to the coast for each taxon, determined by calculating the Euclidean distance between the geographic range centroid (geometric center) and the closest coastline. Geographic range maps were obtained from NatureServe [30]. Geographic distance to the coast was transformed using the natural log to meet normality assumptions.

### 4. Statistical Analyses

#### 4.1. Vulnerability assessments (SIVVA)

We tested whether each SIVVA module score (vulnerability, adaptive capacity, conservation value, information availability) differed between endangered and non-endangered subspecies using linear mixed effects models. Each model contained a fixed effect of taxon status (endangered vs. non-endangered) and random effects of assessor identity and taxon-pair. We tested for the effect of taxon status on SIVVA scores by comparing models with and without the fixed effect using a chi-square statistic [31]. Because each taxon was assessed by two independent experts, pairwise differences within taxon-pairs were evaluated using 95% confidence intervals around each SIVVA module score, calculated for each taxon using 1000 Monte Carlo simulations based on scoring uncertainty.

We developed four *a priori* hypotheses regarding vulnerability to sea level rise, habitat fragmentation, altered temperature, and altered precipitation, therefore we also evaluated these vulnerability criteria independently (criteria 1,3,4, and 5 of the vulnerability module; Table S1) using a mixed effects model with fixed and random effects as described for the overall SIVVA analyses. Owing to recent criticisms of the Bonferroni correction including the inflation of type II error and loss of power [32], [33] we accounted for the false discovery rate when conducting multiple tests by controlling for the proportion of false positives among rejected null hypotheses [34], [35]. Scores of the individual vulnerability criteria were non-normal, so we specified a Poisson error distribution in all models except the model for sea level rise. We tested for differences in sea level rise vulnerability using the proportion of habitat lost to 1 m of sea level rise by 2100. The proportion of habitat inundated was calculated via taxon range maps obtained from NatureServe [30]. The sea level rise scenario map was generated using ArcGIS v10 software and a “bathtub inundation” approach based on a 10 m resolution digital elevation map. This scenario represents a modest estimate of sea level rise that will likely occur by 2100 [36], [37], [38]. Because vulnerability to sea level rise was scored as a proportion, we used a logit transformation for these data [39]. The test for vulnerability associated with habitat fragmentation was based on SIVVA criterion scores that assessed potential habitat limitations (including dispersal and migration paths) resulting from natural barriers and human land use practices. This was evaluated using taxon range maps (which included natural features) in conjunction with Florida 2060 population projections and associated forecasts estimating future development and land use changes [40]; this source represented the only accessible statewide projections at the time of this research. To assess variation in vulnerability to altered temperature and precipitation, we compared SIVVA vulnerability criteria scores evaluating taxon dependence on narrow temperature or precipitation regimes. As before, significance of all models was tested by comparing models with and without the fixed effect (endangered vs. non-endangered) using a chi-square statistic [31]. Because each taxon was evaluated by two independent assessors, differences within taxon pairs were evaluated using 95% confidence intervals around each score, as determined from Monte Carlo simulations based on scoring uncertainty.

#### 4.2. Ecological traits

To test for differences in ecological traits between endangered and non-endangered subspecies across all taxon-pairs, trait data were converted to proportions of the maximum value for each taxon-pair x trait combination (which allowed for comparisons across taxon-pairs with data that varied in scale). Proportion data were logit transformed to account for non-normality, and the minimum non-zero proportion in the data set was added to the numerator and denominator of the logit function to correct for proportion values equal to one [39]. Data were analyzed using a linear mixed effects model with trait nested within taxon status (endangered vs. non-endangered); taxon-pair was included as a random effect. Tukey’s HSD contrasts were calculated to determine pairwise differences between all endangered and non-endangered subspecies for each trait. In addition to the ‘global’ analysis across all taxon-pairs (which tested for overall trait differences between endangered and non-endangered subspecies), trait differences within individual taxon-pairs were calculated using Wilcoxon rank sum tests (non-transformed data) for taxon-pair x trait combinations with at least three independent observations per taxon.

Long distance dispersal can affect species range shifts [41], [42], and dispersal capacity may affect the ability to utilize fragmented landscapes [43]. Therefore, we conducted Spearman rank correlations (non-transformed data) between mean and maximum dispersal estimates. Because there was a significant positive correlation between mean and maximum dispersal distance (r = 0.898, p<0.001), and analyses conducted with mean and maximum dispersal metrics produced equivalent results, we chose to report analyses using only mean dispersal distance.

#### 4.3. Relating ecological traits to SIVVA

To determine whether ecological characteristics were associated with SIVVA modules, we conducted Pearson product-moment correlation analyses between ecological traits (transformed data) and both the vulnerability and adaptive capacity SIVVA module scores. Because vulnerability to sea level rise, habitat fragmentation, and altered temperature and precipitation are important in this region, correlation analyses were also conducted between each ecological trait and these four vulnerability criteria. We accounted for the false discovery rate when conducting multiple tests by controlling for the proportion of false positives among rejected null hypotheses [34], [35]. Because vulnerability to habitat fragmentation, altered temperature, and altered precipitation were evaluated on the SIVVA scale from 1–6, we conducted Spearman’s rank correlations for these data, while maintaining Pearson correlations for the percent habitat lost to 1 m of sea level rise.

Statistical analyses were conducted in R [44]. Mixed model analyses were performed using the lme4 package [45], Tukey’s contrasts were calculated using the multcomp package [46], and all other analyses were conducted using the base package in R (α = 0.05 for all analyses).

## Results

### 1. Vulnerability Assessments (SIVVA)

**Figure 1:**
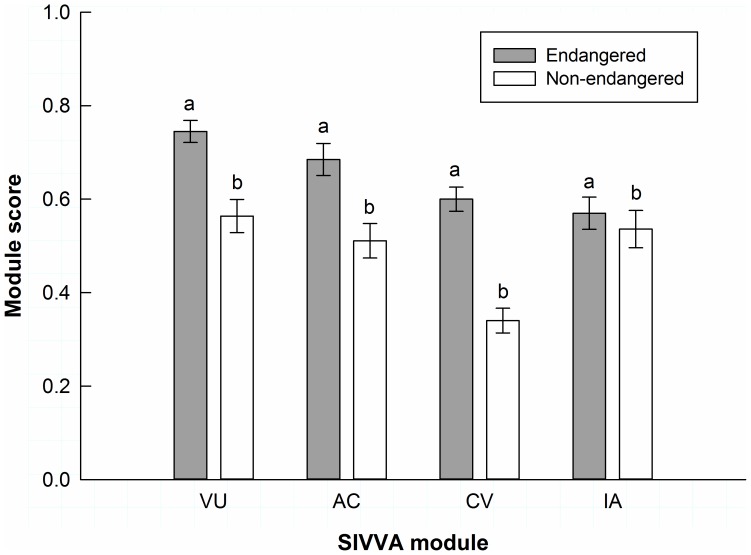
Standardized Index for Vulnerability and Value Assessment (SIVVA) module scores. Mean SIVVA module scores (± SE) for threatened and endangered subspecies (shaded bars) and non-listed subspecies (white bars) for vulnerability (VU), adaptive capacity (AC), conservation value (CV), and information availability (IA). Note that a higher AC module score corresponds to lower adaptive capacity. For each module, bars with different letters are significantly different (p<0.05).

Endangered subspecies as a group had higher SIVVA vulnerability than non-endangered subspecies (χ^2^ = 44.931, df = 1, p<0.001; Figure 1), and pairwise differences within each taxon-pair indicated that all endangered subspecies had significantly higher vulnerability compared to their closely related non-endangered subspecies (p<0.05). For individual vulnerability criteria, endangered subspecies showed greater percent habitat inundation under 1 m of sea level rise (χ^2^ = 13.654, df = 1, p<0.001) and higher vulnerability to habitat fragmentation (χ^2^ = 13.560, df = 1, p<0.001) compared to non-endangered subspecies. Endangered subspecies did not show significantly greater vulnerability to altered temperature (p = 0.559) or precipitation (p = 0.278). Vulnerability to sea level rise, habitat fragmentation, and altered temperature and precipitation for each endangered subspecies are shown in Table 2.

**Table 2:** Threatened and endangered subspecies vulnerability.

	Vulnerability			
Scientific name	Sea level rise	Fragmentation	Temperature	Precipitation
**Mammals**				
*Neotoma floridana smalli*	High	High	Neutral	Neutral
*Odocoileus virginianus clavium*	Very high	Very high	Moderate	Very high
*Oryzomys palustris natator*	Very high	High	Neutral	Neutral
*Puma concolor coryi*	Moderate	High	Moderate	Low
*Peromyscus gossypinus allapaticola*	High	High	Neutral	Neutral
*Peromyscus polionotus niveiventris*	High	Moderate	Neutral	Neutral
*Peromyscus polionotus phasma*	High	High	Neutral	Neutral
*Sylvilagus palustris hefneri*	High	Very high	Moderate	Very high
**Birds**				
*Ammodramus maritimus mirabilis*	High	Very high	Neutral	Neutral
*Ammodramus savannarum floridanus*	Low	Very high	Moderate	Moderate
*Polyborus plancus audubonii*	Low	High	Positive^a^	Neutral
*Rostrhamus sociabilis plumbeus*	Low	High	Moderate	High

Vulnerability of 12 federally threatened and endangered subspecies to sea level rise, habitat fragmentation, altered temperature, and altered precipitation. Sea level rise vulnerability was derived from the percent of habitat inundated under 1 m of sea level rise (Low = 0–25%, Moderate = 26–50%, High = 51–75%, Very high = 76–100%). Vulnerability to habitat fragmentation, altered temperature, and altered precipitation were based on the Standardized Index for Vulnerability and Value Assessment (SIVVA) criteria scores (Neutral = 3, Low = 3–3.75, Moderate = 3.75–4.5, High = 4.5–5.25, Very high = 5.25–6).

a *P. p. audubonii* was evaluated to respond positively to altered temperature.

Endangered subspecies exhibited lower overall adaptive capacity as a group (higher SIVVA adaptive capacity scores) compared to non-endangered subspecies (χ^2^ = 36.436, df = 1, p<0.001; Figure 1). Although each endangered subspecies had lower adaptive capacity than closely related non-endangered subspecies (higher adaptive capacity score), pairwise differences within each taxon-pair indicated this was significantly lower for only 6 out of 12 endangered subspecies (p<0.05). Conservation value was higher for endangered subspecies as a group (χ^2^ = 71.519, df = 1, p<0.001; Figure 1), and pairwise differences indicated that all endangered subspecies had significantly higher conservation value than their non-endangered subspecies pair (p<0.05). Endangered subspecies in our study group also had slightly greater information availability compared with non-endangered subspecies (χ^2^ = 4.207, df = 1, p = 0.040; Figure 1), and pairwise differences showed that 4 out of 12 endangered subspecies had greater information availability.

### 2. Ecological Traits

**Figure 2:**
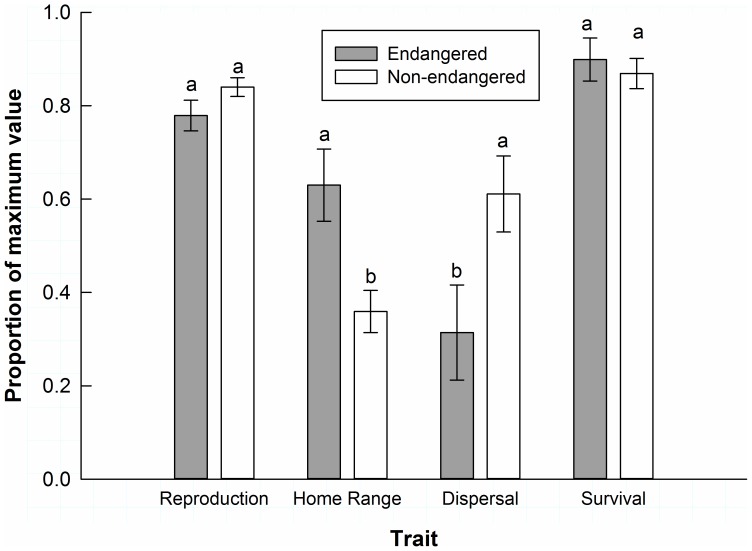
Ecological trait values. Mean ecological trait values (± SE) in threatened and endangered subspecies (shaded bars) and non-listed subspecies (white bars) for litter or clutch size, home range size, dispersal distance, and annual adult survival. Data were transformed to proportions of the maximum value for each taxon-pair x trait combination. For each trait, bars with different letters are significantly different (p<0.05).

Across all taxon-pairs, we detected significant variation among traits (χ^2^ = 80.671, df = 6, p<0.001), and Tukey’s HSD contrasts indicated that endangered subspecies had larger home range sizes (p = 0.002) and shorter dispersal distances (p = 0.033) than non-endangered subspecies (Figure 2). There was no difference in reproductive output per reproductive event or annual adult survival between endangered and non-endangered subspecies across all taxon-pairs.

**Figure 3:**
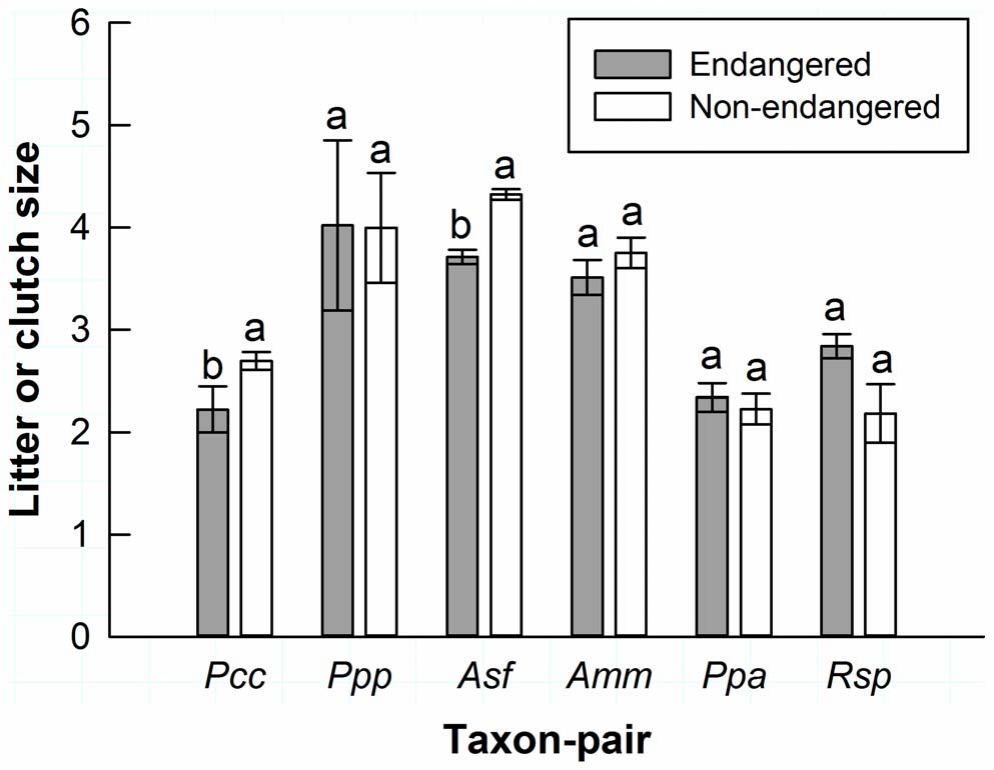
Reproduction within taxon-pairs. Mean litter or clutch size (± SE) for taxon-pairs with a minimum of three independent observations per taxon. For each taxon-pair, bars with different letters are significantly different (shaded bars = threatened and endangered subspecies; white bars = non-listed subspecies; p<0.05). Taxon-pairs are labeled according to the federally listed subspecies: *Pcc* = *Puma concolor coryi*, *Ppp* = *Peromyscus polionotus phasma*, *Asf* = *Ammodramus savannarum floridanus*, *Amm* = *Ammodramus maritimus mirabilis*, *Ppa* = *Polyborus plancus audubonii*, *Rsp* = *Rostrhamus sociabilis plumbeus*.

**Figure 4:**
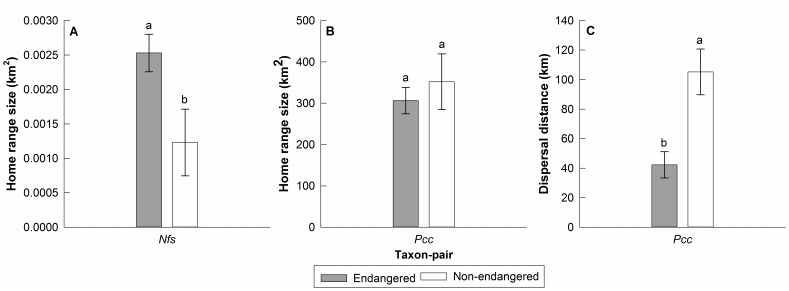
Home range and dispersal within taxon-pairs. Mean home range size and dispersal distance (± SE) for taxon-pairs with a minimum of three independent observations per taxon. Taxon-pairs are labeled according to the federally listed subspecies. (**A**) home range for the *Nfs = Neotoma floridana smalli* taxon-pair, (**B**) home range and (**C**) dispersal distance for the *Pcc* = *Puma concolor coryi* taxon-pair. For each taxon-pair x trait combination, bars with different letters are significantly different (shaded bars = threatened and endangered subspecies; white bars = non-listed subspecies; p<0.05).

Within taxon-pairs, *Puma concolor coryi* (W = 6, n_1_ = 3, n_2_ = 12, p = 0.048) and *Ammodramus savannarum floridanus* (W = 0, n_1_ = 3, n_2_ = 4, p = 0.029) had lower reproductive output than their closely related non-endangered subspecies (Figure 3). Reproductive output did not differ in the other four taxon-pairs with sufficient data to conduct within-taxon-pair comparisons (Figure 3). For the two taxon-pairs with sufficient home range information to conduct within-taxon-pair comparisons, *Neotoma floridana smalli* had a larger home range than *Neotoma floridana floridana* (W = 9, n_1_ = n_2_ = 3, p = 0.050), while home range size of *P. c. coryi* did not differ from other cougars in North America (*Puma concolor couguar*; Table 1; Figure 4) [47]. For dispersal distance there was only one taxon-pair with sufficient information, whereby *P. c. coryi* showed shorter dispersal than *P. c. couguar* (W = 0, n_1_ = 3, n_2_ = 8, p = 0.006; Figure 4). Adequate data was not available to conduct within-taxon-pair comparisons for survival.

### 3. Relating Ecological Traits to SIVVA

**Figure 5:**
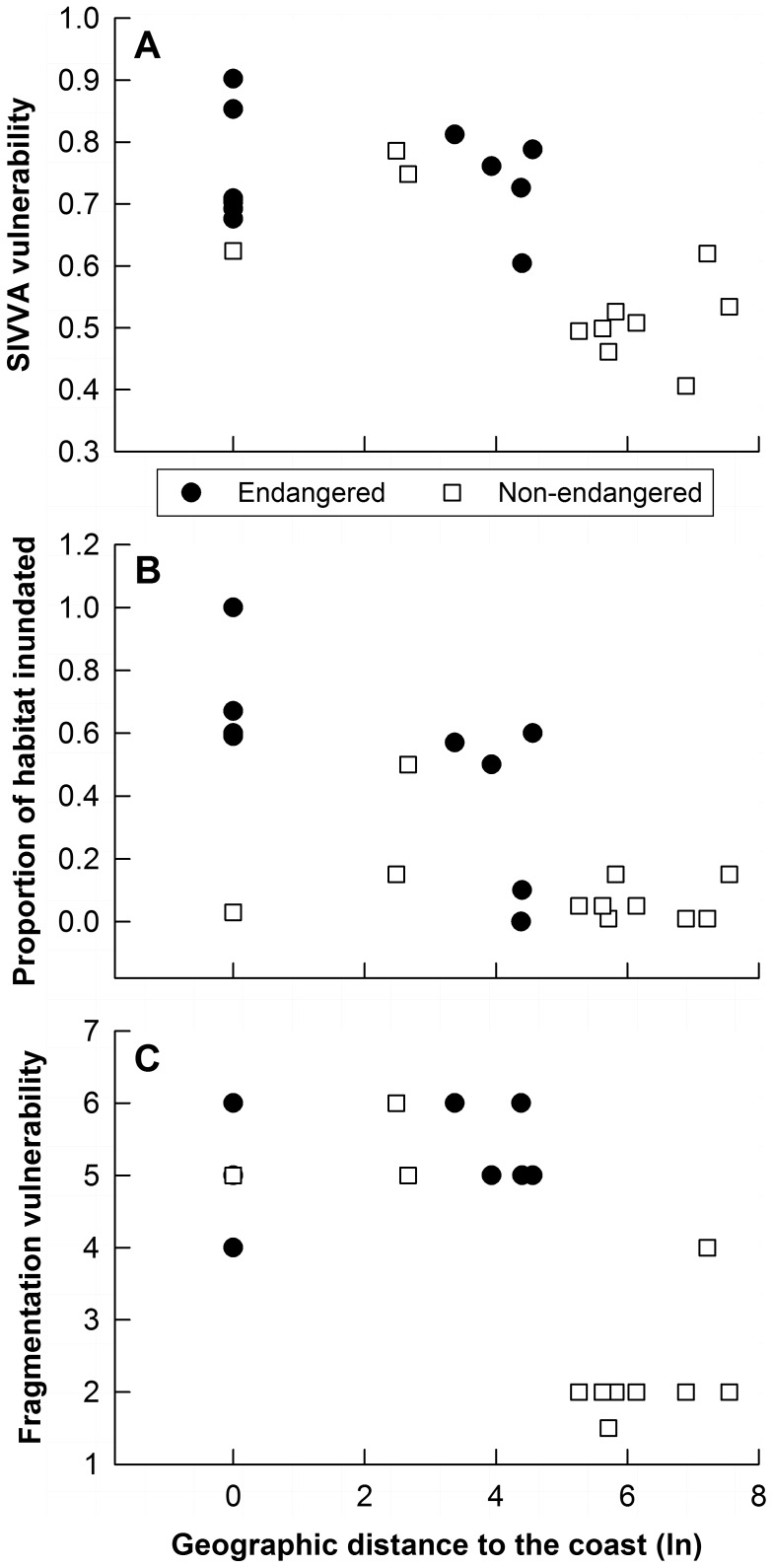
Associations between geographic distance to the coast and vulnerability. Correlation between geographic distance to the coast (natural log) and (**A**) the vulnerability module of the Standardized Index for Vulnerability and Value Assessment (SIVVA) (r = −0.842, p<0.001), (**B**) the proportion of habitat inundated under 1 m of sea level rise (r = −0.675, p<0.001), and (**C**) vulnerability to habitat fragmentation (r = −0.672, p<0.001). Black circles = threatened and endangered subspecies; white squares = non-listed subspecies.

There was no significant correlation between most of the ecological traits and the vulnerability or adaptive capacity SIVVA modules. Not surprisingly, there was a negative correlation between geographic distance to the coast and vulnerability, such that taxa closer to the coast showed higher vulnerability (r = −0.842, p<0.001; Figure 5). We also observed associations between some of the ecological traits and the four *a priori* vulnerability criteria important in this region, where geographic distance to the coast was negatively correlated to both the percent habitat lost to sea level rise (r = −0.675, p<0.001) and habitat fragmentation vulnerability (r = −0.672, p<0.001; Figure 5), indicating that taxa located closer to coast are more susceptible to seal level rise and habitat fragmentation effects. There was a negative correlation between dispersal distance and vulnerability to altered precipitation (r = −0.880, p = 0.021), where taxa with shorter dispersal also showed greater vulnerability to changes in precipitation.

## Discussion

The high threat of habitat inundation from sea level rise for endangered subspecies is of primary concern because approximately 10% of Florida’s land area lies less than 1 m above current sea level. The mean percentage of habitat loss under a 1 m sea level rise scenario was 52% for endangered subspecies, compared to 11% for non-endangered subspecies. Additionally, all but one endangered subspecies showed high vulnerability to habitat fragmentation (*P*. *p*. *niveiventris* showed moderate vulnerability). Florida also has high rates of human population growth [20] and land use conversion [48]. Sea level rise vulnerability coupled with landscape fragmentation may limit habitat availability and inhibit dispersal into new areas, and highlights the challenge of managing human land use activities in conjunction with biodiversity conservation [49], [50], [51].

Subspecies with the highest proportion of habitat inundation from sea level rise were located in the Florida Keys (*Oryzomys palustris natator*, *Odocoileus virginianus clavium*, *Sylvilagus palustris hefneri*, *Peromyscus gossypinus allapaticola*, *N*. *f*. *smalli*) or in coastal areas (*P*. *p*. *phasma*, *P*. *p*. *niveiventris*). Subspecies in the Florida Keys facing habitat inundation are particularly vulnerable to dispersal limitations imposed by both the island archipelago and human development. Similarly, the entire range of *P*. *p*. *phasma* is located on Anastasia Island, and rising sea levels may exacerbate dispersal limitations because of the limited size of the island and its separation from the mainland. More inland species located in peninsular Florida, such as *Ammodramus maritimus mirabilis* and *P*. *c*. *coryi*, also exhibited relatively high levels of percent habitat lost to 1 m of sea level rise (showing 57% and 50%, respectively), indicating that sea level rise vulnerability is not only an issue for taxa located in coastal areas.

In addition to high vulnerability, endangered subspecies exhibited lower adaptive capacity and higher conservation value compared to non-endangered subspecies. Subspecies with significantly lower adaptive capacity were located in the Florida Keys (*O*. *v*. *clavium*, *N*. *f*. *smalli*, *P*. *g*. *allapaticola*), coastal areas (*P*. *p*. *phasma*, *P*. *p*. *niveiventris*), and central Florida (*A*. *s*. *floridanus*). These taxa not only have greater vulnerability (exposure+sensitivity), but also a compromised ability to adapt to environmental change (adaptive capacity). The higher conservation value observed in the endangered subspecies was attributable to phylogenetic and geographic distinctiveness, endemism, and their endangered status. Even when the endangered status criterion was removed, all endangered subspecies retained their higher conservation value. Additionally, many of the endangered subspecies were rated by experts as having a low probability of recovery (e.g., *P*. *c*. *coryi*, *O*. *v*. *clavium*, *A*. *s*. *floridanus*, *A*. *m*. *mirabilis*, *Polyborus plancus audubonii*, *S*. *p*. *hefneri*, *P*. *p*. *niveiventris*; conservation value module, criterion 7). The assessment of conservation priority for these and other taxa in Florida is extremely valuable because there are limited resources available for conservation efforts.

Trait-based filtering operates when abiotic or biotic factors exclude species with certain ecological traits from a community, while allowing species with other traits to exist [52]. In the context of environmental change (e.g., climate or land use), species with life history traits that make them more sensitive or less adaptable to change are more vulnerable to extinction. The ecological trait data considered in our study helps explain differences in extinction risk, where endangered subspecies showed larger home range sizes and greater dispersal limitation compared to non-endangered subspecies. Larger home range size is often associated with greater extinction risk [53] because it increases sensitivity to fragmentation and exposure to human exploitation or persecution. Larger home range size may be indicative of higher resource requirements or lower habitat quality for endangered subspecies in Florida, which may increase vulnerability. Lower dispersal capacity in endangered subspecies could limit their ability to track changing climate and colonize new areas (e.g., in response to rising sea levels). The compromised life history traits in the endangered subspecies may represent inherent characteristics in these taxa or may be a consequence of the degraded landscape. In either case, it may affect their ability to adapt to rapid environmental change, especially given that many endangered taxa in Florida have already experienced environmental pressures such as habitat degradation and fragmentation.

The overall trend of larger home range size in endangered subspecies was driven by greater home range and territory sizes in small mammals (*N*. *f*. *smalli*, *O*. *p*. *natator*) and sparrows (*A*. *m*. *mirabilis*, *A*. *s*. *floridanus*), which showed a high magnitude of difference between endangered and non-endangered subspecies. The endangered large mammals (*P*. *c*. *coryi*, *O*. *v*. *clavium*) had smaller home ranges than non-endangered subspecies, likely due to habitat fragmentation effects, but the magnitude of difference was smaller. For the taxa with sufficient data to conduct within taxon-pair-comparisons, *N*. *f*. *smalli* showed a larger home range than its non-endangered subspecies, and the home range size *P*. *c*. *coryi* did not differ from its non-endangered subspecies.

All of the endangered subspecies included in the dispersal analyses were more dispersal limited than their closely related non-endangered subspecies, however there was enough data to conduct only one within-taxon-pair comparison, whereby *P. c. coryi* exhibited a shorter dispersal distance than *P. c. couguar*. This is of particular concern because the current geographic range of *P*. *c*. *coryi* is a small fragment of a once widespread distribution [54], which is characterized by several barriers to dispersal, including the Caloosahatchee River to the north, extensive mangrove swamps to the south, and urban development to the east and west [55]. Several males were recently documented north of the Caloosahatchee River, but no females have been recorded in central Florida since the early 1970s [56].

Because there were no overall differences across all taxon-pairs in reproduction or survival between endangered and non-endangered subspecies, these may not be crucial factors affecting vulnerability for most of our study taxa. However, the lower reproductive output in *P*. *c*. *coryi* and *A*. *s*. *floridanus* may indicate these subspecies are more vulnerable to environmental change, because slow life history (e.g., small litter size, long gestation time) is associated with greater extinction risk [57], and reproductive output affects population growth and recovery from disturbance. Due to limited data, we did not incorporate survival rates at earlier life stages (e.g., hatchling, juvenile), although these factors may be crucial for long-term population persistence.

The negative association between geographic distance to the coast and overall vulnerability has implications for conservation, because endangered taxa situated closer to the coast also showed greater habitat inundation under 1 m of sea level rise and greater vulnerability to habitat fragmentation. High population growth [20] and coastal development in this region [58] will affect conservation success for coastal and near-coastal taxa. Our results are consistent with recent studies highlighting the vulnerability of Florida’s coastal species to sea level rise [59], [60], [61]. Additionally, the negative correlation between dispersal distance and vulnerability to changes in precipitation may limit the ability of some taxa to track changing climate (e.g., *P*. *c*. *coryi*, *O*. *v*. *clavium*, *R*. *s*. *plumbeus*) if they need to disperse to find more suitable environments.

This study highlights the importance of assessing ecological traits of endangered subspecies, because roughly 50% of the target group was not included in the study owing to limited availability of information. Although we found slightly greater information availability for endangered subspecies in 4 out of 12 taxon-pairs that were included in the study, we observed a general trend of limited data to conduct within-taxon-pair comparisons. Our framework allowed for high levels of phylogenetic control, especially given that each taxon-pair was comprised of non-interacting closely related taxa, with preference given to those with greatest geographic proximity and similarity. Therefore, despite data limitations, we were able to provide assessments of vulnerability and adaptive capacity of species that often have limited information, are not available for controlled experiments, and are in need of conservation. Our ecological trait assessment along with SIVVA provided a robust approach to evaluating species attributes important for conservation.

Many taxa face multiple interacting threats, which are important to evaluate jointly. The use of SIVVA in conjunction with detailed literature surveys of ecological traits provides an in-depth approach to understand the potential drivers of vulnerability and adaptive capacity, differentiate relative impacts of different threats, and prioritize conservation efforts. This type of approach is crucial to help understand the loss of biodiversity and create conservation management practices that attain the most successful outcomes. According to a recent global assessment, the current rate of biodiversity loss is estimated between 100 and 1000 times greater than the historical or “background” rate, and is attributable to human activities such as land use change [62]. The evaluation of species vulnerability and adaptive capacity is extremely valuable given the importance of biodiversity in ecosystem functioning [62], [63] and sustaining human populations [62]. Studies with more narrow taxonomic or regional focus are more informative for practical conservation [64], [65], and the flexible and modular nature of SIVVA is applicable to a broad range of taxonomic groups and ecological systems. The higher vulnerability and lower adaptive capacity of endangered subspecies in Florida is of particular concern in this region, especially given that many of these subspecies have larger home range sizes and shorter dispersal distances compared to their closely related non-endangered subspecies. The inevitable threat of sea level rise and continued habitat fragmentation in this region underscore the need to create and implement conservation plans that maximize conservation success.

## Acknowledgments

We thank numerous species experts and staff at the Florida Natural Areas Inventory for valuable help with SIVVA assessments. We also thank C. Speroterra and D. N. Bucklin for providing comments on a previous version of this manuscript.

## Supporting Information

Table S1 **List of criteria in each module for the Standardized Index for Vulnerability and Value Assessment (SIVVA).**
(DOCX)

Dataset S1 **Ecological trait data for threatened and endangered subspecies and their closely related non-listed subspecies. Subspecies common name, taxon status (endangered vs. non-endangered), subspecies.**
(XLSX)

## References

[1] Brooks TM, Mittermeier RA, Mittermeier CG, Da Fonseca GAB, Rylands AB, et al (2002) Habitat loss and extinction in the hotspots of biodiversity. Conserv Biol 16: 909–923.

[2] McGeoch MA, Butchart SHM, Spear D, Marais E, Kleynhans EJ, et al (2010) Global indicators of biological invasion: species numbers, biodiversity impact and policy responses. Divers Distrib 16: 95–108.

[3] Smith KF, Acevedo-Whitehouse K, Pedersen AB (2009) The role of infectious disease in biological conservation. Anim Conserv 12: 1–12.

[4] Loehle C, Eschenbach W (2012) Historical bird and terrestrial mammal extinction rates and causes. Divers Distrib 18: 84–91.

[5] Intergovernmental Panel on Climate Change (2007) Climate Change 2007: Synthesis Report. Contribution of Working Groups I, II and III to the Fourth Assessment Report of the Intergovernmental Panel on Climate Change. Core Writing Team: Pachauri RK, Reisinger A, editors. Geneva: Intergovernmental Panel on Climate Change.

[6] Parmesan C (2006) Ecological and evolutionary responses to recent climate change. Annu Rev Ecol Evol Syst 37: 637–669.

[7] Parmesan C, Yohe G (2003) A globally coherent fingerprint of climate change impacts across natural systems. Nature 421: 37–42. 12511946 10.1038/nature01286

[8] Root TL, Price JT, Hall KR, Schneider SH, Rosenzweig C, et al (2003) Fingerprints of global warming on wild animals and plants. Nature 421: 57–60. 12511952 10.1038/nature01333

[9] Knight GR, Oetting JB, Cross L (2011) Atlas of Florida’s Natural Heritage – Biodiversity, Landscapes, Stewardship, and Opportunities. Tallahassee: Florida State University.

[10] Stein BA, Kutner LS, Adams JS (2000) Precious Heritage: The Status of Biodiversity in the United States. Oxford: Oxford University Press.

[11] U.S. Fish and Wildlife Service (2012) U.S. Fish & Wildlife Service endangered species program. http://www.fws.gov/endangered/species/index.html. Accessed 2012 July 12.

[12] Weiss J, Overpeck J (2003) Maps of areas susceptible to sea level rise. http://www.geo.arizona.edu/dgesl/research/other/climate_change_and_sea_level/sea_level_rise/sea_level_rise.htm. Accessed 2012 April 15.

[13] Ross MS, O’Brien JJ, Ford RG, Zhang K, Morkill A (2009) Disturbance and the rising tide: the challenge of biodiversity management on low-island ecosystems. Front Ecol Environ 7: 471–478.

[14] Zhang K, Dittmar J, Ross M, Bergh C (2011) Assessment of sea level rise impacts on human population and real property in the Florida Keys. Clim Change 107: 129–146.

[15] Noss RF (2011) Between the devil and the deep blue sea: Florida’s unenviable position with respect to sea level rise. Clim Change 107: 1–16.

[16] Christensen JH, Hewitson B, Busuioc A, Chen A, Gao X, et al. (2007) 2007: regional climate projections. In: Solomon S, Qin D, Manning M, Chen Z, Marquis M, et al.., editors. Climate Change 2007: The Physical Science Basis. Contribution of Working Group I to the Fourth Assessment Report of the Intergovernmental Panel on Climate Change. Cambridge and New York: Cambridge University Press. 847–940.

[17] Von Holle B, Wei Y, Nickerson D (2010) Climatic variability leads to later seasonal flowering of Floridian plants. PLoS ONE 5: 1–9. 10.1371/journal.pone.0011500 PMC2908116 20657765

[18] Misra V, Carlson E, Craig RK, Enfield D, Kirtman B, et al (2011) Climate scenarios: a Florida-centric view. Florida Climate Change Task Force. Center for Ocean-Atmospheric Prediction Studies 14: 1–61.

[19] Parmesan C, Root TL, Willig MR (2000) Impacts of extreme weather and climate on terrestrial biota. B Am Meteorol Soc 81: 443–450.

[20] Mackun P, Wilson S (2011) Population Distribution and Change: 2000 to 2010. U.S. Department of Commerce, Economics and Statistics Administration, U.S. Census Bureau C2010BR-01.

[21] Pimentel D, Zuniga R, Morrison D (2005) Update on the environmental and economic costs associated with alien-invasive species in the United States. Ecol Econ 52: 273–288.

[22] South Florida Water Management District (2010) Executive summary 2010 south Florida environmental report. West Palm Beach: South Florida Water Management District.

[23] Reece JS, Noss RF (2014) Prioritizing species by conservation value and vulnerability: a new index applied to species threatened by sea-level rise and other risks in Florida. Nat Areas J (In press).

[24] Cardillo M (2003) Biological determinants of extinction risk: why are smaller species less vulnerable? Anim Conserv 6: 63–69.

[25] Fritz SA, Bininda-Emonds ORP, Purvis A (2009) Geographical variation in predictors of mammalian extinction risk: big is bad, but only in the tropics. Ecol Lett 12: 538–549. 19392714 10.1111/j.1461-0248.2009.01307.x

[26] Massot M, Clobert J, Ferriere R (2008) Climate warming, dispersal inhibition and extinction risk. Glob Change Biol 14: 461–469.

[27] Integrated Taxonomic Information System (2012) Integrated Taxonomic Information System. http://www.itis.gov/index.html. Accessed 2012 September 14.

[28] Clevenger AP, Wierzchowski J, Chruszcz B, Gunson K (2002) GIS-generated, expert-based models for identifying wildlife habitat linkages and planning mitigation passages. Conserv Biol 16: 503–514.

[29] Martin TG, Burgman MA, Fidler F, Kuhnert PM, Low-Choy S, et al (2012) Eliciting expert knowledge in conservation science. Conserv Biol 26: 29–38. 22280323 10.1111/j.1523-1739.2011.01806.x

[30] NatureServe (2010) NatureServe: A network connecting science with conservation. www.natureserve.org. Accessed 2012 April 10.

[31] Bolker BM, Brooks ME, Clark CJ, Geange SW, Poulson JR, et al (2009) Generalized linear mixed models: a practical guide for ecology and evolution. Trends Ecol Evol 24: 127–135. 19185386 10.1016/j.tree.2008.10.008

[32] Moran MD (2003) Arguments for rejecting the sequential Bonferroni in ecological studies. Oikos 100: 403–405.

[33] Nakagawa S (2004) A farewell to Bonferroni: the problems of low statistical power and publication bias. Behav Ecol 15: 1044–1045.

[34] Benjamini Y, Yekutieli D (2001) The control of the false discovery rate in multiple testing under dependency. Ann Stat 29: 1165–1188.

[35] García LV (2004) Escaping the Bonferroni iron claw in ecological studies. Oikos 105: 657–663.

[36] Pfeffer WT, Harper JT, O’Neel S (2008) Kinematic constraints on glacier contributions to 21^st^-century sea-level rise. Science 321: 1340–1343. 18772435 10.1126/science.1159099

[37] Strauss BH, Ziemlinski R, Weiss JL, Overpeck JT (2012) Tidally adjusted estimates of topographic vulnerability to sea level rise and flooding for the contiguous United States. Environ Res Lett 7: 014033.

[38] Vermeer M, Rahmstorf S (2009) Global sea level linked to global temperature. Proc Natl Acad Sci USA 106: 21527–21532. 19995972 10.1073/pnas.0907765106 PMC2789754

[39] Warton DI, Hui FKC (2011) The arcsine is asinine: the analysis of proportions in ecology. Ecology 92: 3–10. 21560670 10.1890/10-0340.1

[40] Zwick PD, Carr MH (2006) Florida 2060: a population distribution scenario for the state of Florida. Gainesville: Geoplan Center at the University of Florida.

[41] Pearson RG, Dawson TP (2005) Long-distance plant dispersal and habitat fragmentation: identifying conservation targets for spatial landscape planning under climate change. Biol Conserv 123: 389–401.

[42] Phillips BL, Brown GP, Travis JM, Shine R (2008) Reid’s paradox revisited: the evolution of dispersal kernels during range expansion. Am Nat 172: S34–S48. 18554142 10.1086/588255

[43] Van Teeffelen AJA, Vos CC, Opdam P (2012) Species in a dynamic world: consequences of habitat network dynamics on conservation planning. Biol Conserv 153: 239–253.

[44] R Development Core Team (2012) R: A language and environment for statistical computing. http://www.R-project.org. Accessed 2011 December 1. Vienna: R Foundation for Statistical Computing.

[45] Bates D, Maechler M, Bolker B (2012) lme4: Liner mixed-effects models using S4 classes. R package version 0.999999-0. http://CRAN.R-project.org/package=lme4. Accessed 2012 January 4.

[46] Hothorn T, Bretz F, Westfall P (2008) Simultaneous inference in general parametric models. Biom J 50: 346–363. 18481363 10.1002/bimj.200810425

[47] Culver M, Johnson WE, Pecon-Slattery J, O’Brien SJ (2000) Genomic ancestry of the American puma (*Puma concolor*). J Hered 91: 186–197. 10833043 10.1093/jhered/91.3.186

[48] Walker RT, Solecki WD (1997) Land use dynamics and ecological transition: the case of south Florida. Urban Ecosyst 1: 37–47.

[49] Brussaard L, Caron P, Campbell B, Lipper L, Mainka S, et al (2010) Reconciling biodiversity conservation and food security: scientific challenges for a new agriculture. Environ Sustain 2: 34–42.

[50] Noss RF, Murphy DD (1995) Endangered species left homeless in Sweet Home. Conserv Biol 9: 229–231.

[51] Tscharntke T, Clough Y, Wanger TC, Jackson L, Motzke I, et al (2012) Global food security, biodiversity conservation and the future of agricultural intensification. Biol Conserv 151: 53–59.

[52] Weiher E, Keddy PA (1995) Assembly rules, null models, and trait dispersion – new questions from old patterns. Oikos 74: 159–164.

[53] Woodroofe R, Ginsberg JR (1998) Edge effects and the extinction of populations inside protected areas. Science 280: 2126–2128. 9641920 10.1126/science.280.5372.2126

[54] Kautz R, Kawula R, Hoctor T, Comiskey J, Jansen D, et al (2006) How much is enough? Landscape-scale conservation for the Florida panther. Biol Conserv 130: 118–133.

[55] Thatcher CA, van Manen FT, Clark JD (2006) Identifying suitable sites for Florida panther reintroduction. J Wildl Manage 70: 752–763.

[56] Thatcher CA, van Manen FT, Clark JD (2009) A habitat assessment for Florida panther population expansion into central Florida. J Mammal 90: 918–925.

[57] Purvis A, Gittleman JL, Cowlishaw G, Mace GM (2000) Predicting extinction risk in declining species. Proc R Soc Lond B Biol Sci 267: 1947–1952. 10.1098/rspb.2000.1234 PMC1690772 11075706

[58] Finkl CW, Charlier RH (2003) Sustainability of subtropical coastal zones in southeastern Florida: challenges for urbanized coastal environments threatened by development, pollution, water supply, and storm hazards. J Coast Res 19: 934–943.

[59] Maschinski J, Ross MS, Liu H, O’Brien JO, von Wettberg EJ, et al (2011) Sinking ships: conservation options for endemic taxa threatened by sea level rise. Clim Change 107: 147–167.

[60] Saha AK, Saha S, Sadle J, Jiang J, Ross MS, et al (2011) Sea level rise and South Florida coastal forests. Clim Change 107: 81–108.

[61] Schmidt JA, McCleery R, Seavey JR, Cameron Devitt SE, Schmidt PM (2012) Impacts of a half century of sea-level rise and development on an endangered mammal. Glob Change Biol 18: 3536–3542.

[62] Rockström J, Steffen W, Noone K, Persson Å, Chapin III FS, et al (2009) A safe operating space for humanity. Nat 461: 472–475. 10.1038/461472a 19779433

[63] Naeem S, Duffy JE, Zavaleta E (2012) The functions of biological diversity in an age of extinction. Science 336: 1401–1406. 22700920 10.1126/science.1215855

[64] Cardillo M, Georgina MM, Gittleman JL, Jones KE, Bielby J, et al (2008) The predictability of extinction: biological and external correlates of decline in mammals. Proc R Soc Lond B Biol Sci 275: 1441–1448. 10.1098/rspb.2008.0179 PMC2602711 18367443

[65] Fisher DO, Owens IPF (2004) The comparative method in conservation biology. Trends Ecol Evol 19: 391–398. 16701291 10.1016/j.tree.2004.05.004

[66] Indorf JL (2010) Phylogeography of the marsh rice rat (*Oryzomys palustris*) in wetlands of the southeastern United States. Doctor of philosophy dissertation. Coral Gables: University of Miami.

