# Supplementary material for: Threatened and Endangered Subspecies with Vulnerable Ecological Traits Also Have High Susceptibility to Sea Level Rise and Habitat Fragmentation
Source: PLoS One. 2013 Aug 5;8(8):e70647. doi: 10.1371/journal.pone.0070647 (PMC3734267; doi:10.1371/journal.pone.0070647)
Supplement: Table S1 — List of criteria in each module for the Standardized Index for Vulnerability and Value Assessment (SIVVA). (DOCX) [file pone.0070647.s001.docx]

**TableS1.** **List of criteria in each module for the Standardized Index for Vulnerability and Value Assessment (SIVVA)**.

| Module | Criterion |
| --- | --- |
| Vulnerability | 1 Proportion of habitat inundated by sea level rise |
|  | 2 Habitat susceptibility to erosion from sea level rise |
|  | 3 Vulnerability of habitat/dispersal corridors to fragmentation |
|  | 4 Vulnerability to altered temperature |
|  | 5 Vulnerability to altered precipitation |
|  | 6 Portion of range that is protected |
|  | 7 Are populations of the species highly disjunct? |
|  | 8 Vulnerability to increased salinity from sea level rise |
|  | 9 Vulnerability to storm surge or run-off |
|  | 10 Vulnerability to altered biotic interactions |
|  | 11 Synergistic effects of sea level rise, human development, and climate change |
|  | 12 Vulnerability to changes in natural disturbance regime |
| Adaptive capacity | 1 Ability to disperse/migrate in response to threats |
|  | 2 Amount of phenotypic plasticity |
|  | 3 Amount of genetic diversity |
|  | 4 Adaptive rate (i.e., generation time, fecundity) |
|  | 5 Demographic capacity to adapt or migrate |
|  | 6 Colonization potential |
| Conservation value | 1 Level of endemism |
|  | 2 Level of separation in populations/lineages |
|  | 3 Is it a keystone or foundation species? |
|  | 4 Level of phylogenetic distinctiveness |
|  | 5 Level of economic value |
|  | 6 Level of endangerment based of state or federal listing |
|  | 7 Probability of recovery success |
| Information availability | 1 Amount of published literature |
|  | 2 Number of demographic/niche models |
|  | 3 Amount population genetic data available |
|  | 4 Amount of information on sea level rise responses |
|  | 5 Amount of information on climate change responses |

There are four modules and 30 total criteria in SIVVA (n = 12 vulnerability criteria, n = 6 adaptive capacity criteria, n = 7 conservation value criteria, n = 5 information availability criteria).
